# Serum proteome signatures associated with liver steatosis in adolescents with obesity

**DOI:** 10.1007/s40618-024-02419-x

**Published:** 2024-07-17

**Authors:** P. J. Giraudi, D. Pascut, C. Banfi, S. Ghilardi, C. Tiribelli, A. Bondesan, D. Caroli, A. Minocci, A. Sartorio

**Affiliations:** 1 Metabolic Liver Disease Unit, Fondazione Italiana Fegato-ONLUS, Trieste, Italy; 2 Liver Cancer Unit, Fondazione Italiana Fegato-ONLUS, Trieste, Italy; 3 Unit of Functional Proteomics, Metabolomics, and Network Analysis, Centro Cardiologico Monzino, IRCCS, Milan, Italy; 4 Istituto Auxologico Italiano IRCCS, Experimental Laboratory for Auxo-endocrinological Research, Piancavallo-Verbania, Italy; 5 Division of Metabolic Diseases, Istituto Auxologico Italiano IRCCS, Piancavallo-Verbania, Italy; 6 Istituto Auxologico Italiano IRCCS, Experimental Laboratory for Auxo-endocrinological Research, Milan, Italy

**Keywords:** Proteomics, Pediatrics, Circulating biomarkers, Steatosis, Cardiovascular, Metabolic, MAFLD

## Abstract

**Purpose:**

Childhood obesity, a pressing global health issue, significantly increases the risk of metabolic complications, including metabolic dysfunction associated with steatotic liver disease (MASLD). Accurate non-invasive tests for early detection and screening of steatosis are crucial. In this study, we explored the serum proteome, identifying proteins as potential biomarkers for inclusion in non-invasive steatosis diagnosis tests.

**Methods:**

Fifty-nine obese adolescents underwent ultrasonography to assess steatosis. Serum samples were collected and analyzed by targeted proteomics with the Proximity Extension Assay technology. Clinical and biochemical parameters were evaluated, and correlations among them, the individuated markers, and steatosis were performed. Receiver operating characteristic (ROC) curves were used to determine the steatosis diagnostic performance of the identified candidates, the fatty liver index (FLI), and their combination in a logistic regression model.

**Results:**

Significant differences were observed between subjects with and without steatosis in various clinical and biochemical parameters. Gender-related differences in the serum proteome were also noted. Five circulating proteins, including Cathepsin O (CTSO), Cadherin 2 (CDH2), and Prolyl endopeptidase (FAP), were identified as biomarkers for steatosis. CDH2, CTSO, Leukocyte Immunoglobulin Like Receptor A5 (LILRA5), BMI, waist circumference, HOMA-IR, and FLI, among others, significantly correlated with the steatosis degree. CDH2, FAP, and LDL combined in a logit model achieved a diagnostic performance with an AUC of 0.91 (95% CI 0.75–0.97, 100% sensitivity, 84% specificity).

**Conclusions:**

CDH2 and FAP combined with other clinical parameters, represent useful tools for accurate diagnosis of fatty liver, emphasizing the importance of integrating novel markers into diagnostic algorithms for MASLD.

**Supplementary Information:**

The online version contains supplementary material available at 10.1007/s40618-024-02419-x.

## Introduction

The escalating prevalence of childhood obesity has emerged as a significant public health concern globally, with profound implications for the development of metabolic complications, including metabolic dysfunction associated with steatotic liver disease (MASLD) [1, 2]. Recent studies have highlighted the alarming prevalence of liver steatosis not only in adolescents with obesity but also in non-obese counterparts, underscoring the need for comprehensive diagnostic approaches across diverse populations [3, 4]. Obesity significantly increases the risk of liver steatosis in adolescents. Up to 45% of obese individuals may show hepatic fat accumulation, detectable by imaging techniques such as ultrasonography [5]. Moreover, while liver steatosis represents an early and reversible stage of MASLD, more advanced forms of the disease, such as metabolic dysfunction associated with steatohepatitis (MASH) and liver fibrosis, pose greater health risks and are associated with increased morbidity and mortality. Indeed, studies have indicated that approximately 20–30% of individuals with liver steatosis progress to MASH, characterized by hepatic inflammation and injury, while a subset of patients may develop liver fibrosis, cirrhosis, and hepatocellular carcinoma over time [6]. In the pediatric population, MAFLD is typically diagnosed between the ages of 10 and 13 years. The actual onset of disease for most children is not known. At the moment of the diagnosis, 10–25% of children can have advanced fibrosis. In the most severe cases, children can progress within a few years to cirrhosis and end-stage liver disease. Quality longitudinal data on the natural history of pediatric MAFLD are limited [7]. This highlights the critical need for accurate and timely diagnosis of liver steatosis, particularly in adolescents with obesity, to mitigate the risk of progression to more severe liver-related complications.

Traditionally, the gold standard for diagnosing liver steatosis has been liver biopsy, a procedure associated with inherent risks and limitations, particularly in pediatric populations [8]. Given the invasiveness and potential for complications associated with liver biopsy, there is growing interest in non-invasive approaches that can accurately assess liver fat content and steatosis severity without the need for tissue sampling [9].

Several non-invasive clinical scores have been developed to diagnose hepatic steatosis based on clinical and laboratory parameters. These include the Fatty Liver Index (FLI) [10], NAFLD Liver Fat Score (NLFS), Hepatic Steatosis Index (HSI) [11, 12], and the Zhe Jiang University Index (ZJU) [13]. Furthermore, other scoring systems utilize various parameters, such as anthropometric measurements, liver function tests, and lipid profiles, to estimate the probability of liver steatosis and fibrosis. Examples include The Pediatric NAFLD Fibrosis Index (PNFI) [14], APRI, and FIB-4 [15].

In addition to clinical scores, a variety of imaging modalities have been employed for non-invasive assessment of liver steatosis, including ultrasound (US), computed tomography (CT), magnetic resonance imaging (MRI), and transient elastography (TE) [16]. Ultrasonography, with its widespread availability, low cost, and lack of ionizing radiation, represents a first-line imaging modality for detecting hepatic steatosis in clinical practice [10, 11].

Nowadays, there is an active interest in the research of serum markers that could represent a great promise for precision medicine by adding a layer of molecular characterization. Proteomic approaches (such as the proximity extension assay (PEA) [12], the Slow Off-rate Modified aptamers assay (SOMAmers) [13], the NUcleic acid Linked ImmunoaSandandwich Assay (NULISA™) [14], among others) have been used to identify protein biomarkers that could be combined with clinical-biochemical variables in non-invasive diagnostic models for steatosis [17], at-risk MASH [18, 19], MASH and fibrosis [17, 20, 21], and cirrhosis [22] in MASLD. However, most studies focus on advanced stages of liver disease, disregarding steatosis, which is often absent in pediatric studies. Therefore, we aimed to explore the serum proteome using the Proximity extension assay (PEA) approach, identifying proteins as potential biomarkers for inclusion in future non-invasive algorithms for diagnosing steatosis in pediatric subjects. Early detection of fatty liver presents an opportunity to address the associated obesity and other metabolic risk factors that may lead to the development of more advanced stages of metabolic-associated fatty liver disease (MASLD) or worsen comorbidities. Additionally, screening tools to identify individuals at risk of MASLD will allow early intervention and risk stratification in subjects with obesity.

## Materials and methods

### Patients

In this observational monocentric study, fifty-nine adolescents with obesity (age range 11–17.9 years; mean ± SD 15.7 ± 1.7 years; BMI SDS range 2.0–4.3, mean BMI SDS 3.1 ± 0.6) calculated according to the age and sex Italian growth charts [23] were enrolled between March 2022 and January 2023. All the subjects were hospitalized at the Division of Auxology, Istituto Auxologico Italiano, Piancavallo-Verbania, Italy for a multidisciplinary body weight reduction program. Blood samples were obtained in the first two days of hospitalization. 22 patients (37%) suffered from metabolic syndrome, based on the IDF (International Diabetes Federation) criteria for pediatric populations [24, 25]; 37 patients (63%) had liver steatosis evaluated by liver ultrasonography (degree 1: 13 degree 2: 9, degree 3: 15), as previously described [26] and according to standardized criteria [27, 28]. Five patients (8.5%) were taking oral anti-diabetics, 2 patients (3.4%) were taking anti-hypertensive drugs, 4 patients (6.7%) were taking antidepressants/anxiolytics, 1 patient (1.7%) was taking levothyroxine, and 2 patients (3.4%) were taking oral contraceptives. The Ethics Committee of Istituto Auxologico Italiano Milan, Italy (ethical committee code: 2022_3_15_08;_research code: 01C217; acronym: POSING) approved the study. All procedures in the study complied with the Helsinki Declaration of 1975, as revised in 2008. The research procedure was explained to each participant, and written informed consent was obtained by subjects and their parents when it was appropriate.

### Assessment of body composition

Body composition was measured by using a multifrequency tetrapolar impedancemeter (BIA, Human-IM Scan, DS-Medigroup, Milan, Italy) with a delivered current of 800 uA at a frequency of 50 kHz. To reduce errors of measurement, attention was paid to the standardization of the variables that affect measurement validity, reproducibility, and precision. Measurements were performed according to the method of [29] after 20 min of resting in a supine position with arms and legs relaxed and not in contact with other body parts. Fat-free mass (FFM) was calculated using the prediction equation [30], and fat mass (FM) was obtained by subtracting FFM from body weight (BW)**.**

### Serum collection for laboratory assessment and proteome analysis

After an overnight fast, blood samples were obtained through standard venipuncture using BD Vacutainer® serum separating tubes (BD—Plymouth PL6 7BP, UK). The tubes were centrifuged at 1900×*g* for 10 min at 4 °C. Then, the resulting supernatants were carefully transferred into new tubes. Laboratory analysis including alanine aminotransferase (ALT), aspartate aminotransferase (AST), gamma-glutamyl transferase (GGT), alkaline phosphatase, total bilirubin, total cholesterol, high-density lipoprotein (HDL) cholesterol, low-density lipoprotein (LDL) cholesterol, triglycerides, and c-reactive protein (CRP) were measured using a Cobas 6000 analyzer (Roche Diagnostics, SPA, Monza, Italy). For proteomics analysis, the samples of the first centrifugation were centrifuged again at 16,000×*g* for 10 min at 4 °C, and the supernatants were aliquoted into new tubes and promptly frozen at -80 °C to ensure long-term storage until analysis.

### Serum proteome profiling and analysis

Serum samples were analyzed with the PEA technology using the Metabolism and Cardiometabolic Olink Target 96 panels (Olink Proteomics, Uppsala, Sweden). Briefly, the target protein binds to the double oligonucleotide-labeled antibody probe with high specificity. Then, the microfluidic real-time PCR amplification of the oligonucleotide sequence is used to detect the resulting DNA sequence quantitatively [26]. Using internal and external controls, the resulting threshold cycle (Ct)-data were processed for quality control and normalized. Protein levels were measured on a relative scale and presented as a normalized protein expression (NPX), an arbitrary unit on a log2 scale. A high NPX value corresponds to a high protein concentration. A list of proteins included in the analysis is shown in Table S1. Data visualization, exploration, and initial statistical analysis were conducted using the Olink Statistical Analysis web-based app OlinKSAA [31]. The NPX dataset was uploaded into the application, and samples that did not meet the quality controls were excluded from the analysis. The samples were then categorized based on the grade of steatosis, and an analysis of variance (ANOVA) was performed on each assay. The results were presented as NPX median values and the inter-quartile range (IQR) for each marker in the sample group unless differently specified. Using the Benjamini–Hochberg method, the reported p-values from the ANOVA analysis were adjusted for multiple testing.

### Bioinformatics Analysis

A protein–protein interaction network (PPI) was obtained for the individuated proteins as candidates for steatosis discrimination using the NetworkAnalyst visual analytics platform [32]. UniprotKb IDs were used to build the PPI, and the Generic PPI combined with Tissue-specific PPI options were selected in the toolbar. IMEx interactome and first-order building options were also applied in the NetworkAnalyst platform. Then, functional enrichment analyses for all PPI components were performed using the Reactome (https://reactome.org/) platform. This process allowed us to gain insights into the functional implications of the identified proteins in the context of liver steatosis. In addition, the expression data of the proteins of interest were investigated in the Human Protein Atlas data portal (http://proteinatlas.org).

### Statistical methods

Continuous variables were expressed as mean ± (standard deviation) or median (interquartile range) and categorical as numbers or percentages. Categorical variables were analyzed using chi-square tests with correction when appropriate. Independent t-test and ANOVA were used for normally distributed continuous variables. Non-parametric tests (Mann–Whitney, and Kruskal–Wallis with posthoc analysis) were applied for continuous variables that failed to pass the D'Agostino&Pearson omnibus normality test. Correlation analysis was performed using Pearson's or Spearman’s correlation coefficients to estimate the association of serum candidates’ levels and several factors of interest. Statistical analysis was performed by using GraphPad Prism 10.2.0. Volcano and correlation matrix plots were performed using Python packages (Python scripts are available in supplementary materials). The diagnostic performance of candidates was assessed by receiver operating characteristic (ROC) curves. The area under the ROC (AUROC) was used to compare the accuracy of different steatosis diagnostic candidates and indexes. The sensitivity, specificity, positive predictive values (PPVs), and negative predictive values (NPVs) for relevant cut-offs were also calculated. A forward selection with switching one-way logistic regression analysis was used to estimate the discriminatory potential of the protein biomarkers in combination with additional clinic-biochemical parameters. ROCs and logistic regression analysis were performed using NCSS 11 Software (2016) (NCSS, LLC. Kaysville, Utah, USA, ncss.com/software/ncss).

## Results

### Morpho-clinic characteristics of the subjects

The cohort under study (*n* = 59) was stratified according to absence (S0) or presence (S1-S2-S3) of liver steatosis. Table 1 shows the clinical characteristics of the enrolled subjects. Significant differences (p < 0.05) between groups were observed for gender, BMI, waist circumference, measured resting energy expenditure (mREE), insulin, HOMA-IR, total and LDL cholesterol, liver transaminases, APRI, and the fatty liver index (FLI). All these parameters were higher in the group with steatosis. Table 2 shows the statistical differences when the cohort was stratified by steatosis and gender. In this case, significant differences inside steatosis groups were obtained for FFM, FM**,** REE in both genders, and liver enzymes, particularly in males (AST, ALT, GGT, and the APRI). Comparing the S0 and the S1-S2-S3 subgroups, the female cohort presented more significant differences in BMI, waist circumference, total and LDL cholesterol, liver enzymes (ALT and GGT), and FLI.

**Table 1:** Clinical characteristics of the studied population

Variable	Obese cohort (*n* = 59)		
	No steatosis (S0, *n* = 22)	Steatosis (S1-S2-S3, *n* = 37)	*p-value*
Age (years)	16 ± 1	16 ± 2	*0.31*
Female gender (n, %)	16, 51%	12, 43%	*0.001*
BMI (kg/m^2^)	36 ± 4	40 ± 6	*0.01*
Waist circumference (cm)	113 ± 11	127 ± 14	*0.0002*
FFM (%)	53 ± 6	53 ± 5	*0.99*
FM (kg)	47 ± 11	53 ± 15	*0.08*
mREE (kcal)	2024 ± 393	2333 ± 418	*0.008*
Systolic pressure (mm Hg)	126 ± 9	130 ± 14	*0.48*
Diastolic pressure (mm Hg)	78 ± 9	80 ± 5	*0.27*
Fasting glucose (mg/dL)	85 ± 6	88 ± 7	*0.25*
Insulin (mU/L)	20 ± 8	31 ± 15	*0.003*
HOMA-IR	4 ± 2	7 ± 3	*0.002*
Total cholesterol (mg/dL)	150 ± 19	166 ± 32	*0.03*
HDL cholesterol (mg/dL)	44 ± 9	43 ± 10	*0.40*
LDL cholesterol (mg/dL)	92 ± 16	108 ± 28	*0.02*
Triglycerides (mg/dL)	105 ± 34	129 ± 50	*0.05*
C-reactive protein	0.47 ± 0.50	0.53 ± 0.46	*0.14*
Platelets	275 ± 58	270 ± 53	*0.76*
AST (U.I./L)	21 ± 17	29 ± 25	*0.007*
ALT (U.I./L)	24 ± 37	43 ± 47	*0.004*
GGT (U.I./L)	15 ± 7	24 ± 14	*0.002*
APRI	0.21 ± 0.23	0.30 ± 0.33	*0.04*
FLI	77 ± 16	91 ± 11	*0.0001*
Steatosis grade (S0, S1, S2, S3—Female /Male)	16, 0, 0, 0 / 6, 0, 0, 0	0, 6, 3, 4 / 0, 7, 6, 11	*0.03*

*BMI* body mass index, *FFM* fat-free mass, *FM* fat mass, *mREE* measured resting energy expenditure, *HOMA-IR* homeostatic model assessment-insulin resistance, *HDL* high-density lipoprotein, *LDL* low-density lipoprotein, *AST* aspartate aminotransferase, *ALT* alanine aminotransferase, *GGT* gamma-glutamyl transferase, *APRI* aspartate aminotransferase to platelet ratio index, *FLI* fatty liver index; steatosis grade 0: S0, steatosis grade 1, 2 and 3: S1-S2-S3. *p* < 0.05 was considered statistically significant vs. respective controls, S0 group was used as the control. Data are shown as mean ± SD for continuous variables, and number (%) for binary variables. t-test was used to test for significant differences within continuous variables that were normally distributed, while Mann–Whitney and Kruskal–Wallis when non-normally distributed. The chi-Squares test was used for categorical variables

**Table 2:** Clinical-biochemical characteristics of the cohort stratified by steatosis and gender

Variable	Steatosis (S0, *n* = 22)			Steatosis (S1-S2-S3, *n* = 37)			*p-value S1-S2-S3 vs S0*	
	Female (*n* = 16)	Male (*n* = 6)	*p- value*	Female (*n* = 12)	Male (*n* = 25)	*p-value*	Female	Male
Age (years)	16 ± 1	16 ± 1	*0.70*	16 ± 2	16 ± 2	*0.50*	*0.90*	*0.80*
BMI (kg/m^2^)	36 ± 4	35 ± 4	*0.60*	41 ± 6	39 ± 6	*0.50*	*0.01*	*0.22*
Waist circumference (cm)	112 ± 12	117 ± 9	*0.40*	125 ± 17	129 ± 12	*0.40*	*0.03*	*0.08*
FFM (%)	49 ± 9	63 ± 6	*0.004*	53 ± 11	66 ± 10	*0.001*	*0.22*	*0.40*
FM (kg)	48 ± 12	44 ± 7	*0.50*	49 ± 13	56 ± 15	*0.22*	*0.13*	*0.10*
mREE (kcal)	1932 ± 374	2316 ± 330	*0.05*	1973 ± 324	2505 ± 344	*0.000*	*0.56*	*0.30*
Insulin (mU/L)	20 ± 8	18 ± 10	*0.50*	20 ± 8	31 ± 15	*0.90*	*0.07*	*0.02*
HOMA-IR	4.3 ± 2	3.7 ± 2	*0.50*	6 ± 4	7 ± 3	*0.70*	*0.08*	*0.02*
Total cholesterol (mg/dL)	148 ± 20	153 ± 20	*0.60*	179 ± 33	160 ± 30	*0.09*	*0.005*	*0.44*
HDL cholesterol (mg/dl)	44 ± 9	39 ± 8	*0.16*	45 ± 12	42 ± 8	*0.27*	*0.95*	*0.50*
LDL cholesterol (mg/dL)	90 ± 17	99 ± 11	*0.30*	118 ± 30	103 ± 27	*0.13*	*0.023*	*0.70*
Triglycerides (mg/dL)	107 ± 38	97 ± 13	*0.30*	135 ± 47	124 ± 52	*0.13*	*0.120*	*0.26*
AST (U.I./L)	21 ± 20	21 ± 9	*0.20*	18 ± 3	34 ± 29	*0.007*	*0.220*	*0.30*
ALT (U.I./L)	23 ± 43	25 ± 11	*0.08*	21 ± 7	53 ± 54	*0.009*	*0.019*	*0.27*
GGT (U.I./L)	12 ± 5	24 ± 8	*0.002*	17 ± 7	27 ± 15	*0.04*	*0.020*	*0.90*
APRI	0.26 ± 0.33	0.25 ± 0.09	*0.15*	0.16 ± 0.03	0.37 ± 0.38	*0.001*	*0.740*	*0.34*
FLI	79 ± 60	59 ± 20	*0.80*	90 ± 15	92 ± 9	*0.80*	*0.007*	*0.09*

*BMI* body mass index, *FFM* fat-free mass, *FM* fat mass, *mREE* measured resting energy expenditure, *HOMA-IR* homeostatic model assessment-insulin resistance, *HDL* high-density lipoprotein, *LDL* low-density lipoprotein, *AST* aspartate aminotransferase, *ALT* alanine aminotransferase; GGT: gamma-glutamyl transferase, *APRI* aspartate aminotransferase to platelet ratio index, *FLI* fatty liver index; steatosis grade 0: S0; steatosis grade 1, 2 and 3: S1-S2-S3. *p* < 0.05 was considered statistically significant vs. respective controls, S0 group was used as the control. Data are shown as mean ± SD for continuous variables, and number (%) for binary variables. t-test was used to test for significant differences within continuous variables that were normally distributed, while Mann–Whitney and Kruskal–Wallis when non-normally distributed. The chi-Squares test was used for categorical variables

### Serum proteins associated with the presence of liver steatosis

**Fig. 1:**
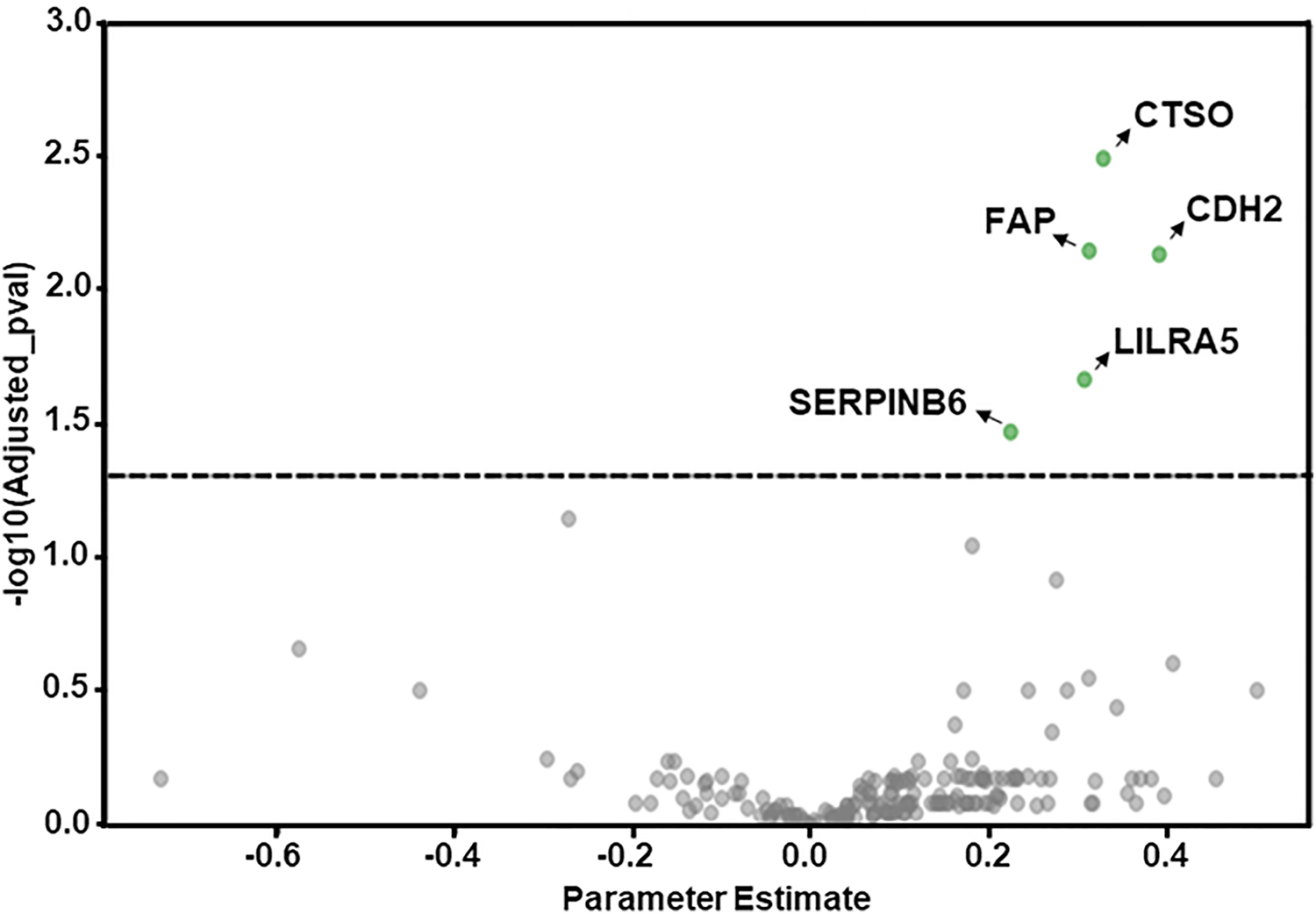
Serum protein expression in obese adolescents with and without steatosis. The volcano plot shows the NPX difference between non-steatotic (*n* = 21) and steatotic (*n* = 37) patients on the x-axis and the –log10 of the nominal *p*-value on the y-axis. All *p*-values were adjusted for multiple testing using the Benjamini–Hochberg method and − log10 of the *p*-values was plotted

The PEA assay for 184 target proteins (metabolic and cardiometabolic panels) was conducted on the 59 serum samples. One sample showed alterations in the internal quality control tests and was removed from subsequent analysis. The study identified five circulating proteins associated with the steatosis grade in the liver: Cathepsin O (CTSO), Cadherin 2 (CDH2), Leukocyte immunoglobulin-like receptor subfamily A member 5 (LILRA5), and Serpin B6 (SERPINB6); and Prolyl endopeptidase (FAP). These proteins showed significant differential abundance when comparing groups with presence vs. absence of steatosis (Fig. 1, Supplementary Figure S1 and Table S2), according to p-values adjusted for multiple testing.

**Fig. 2:**
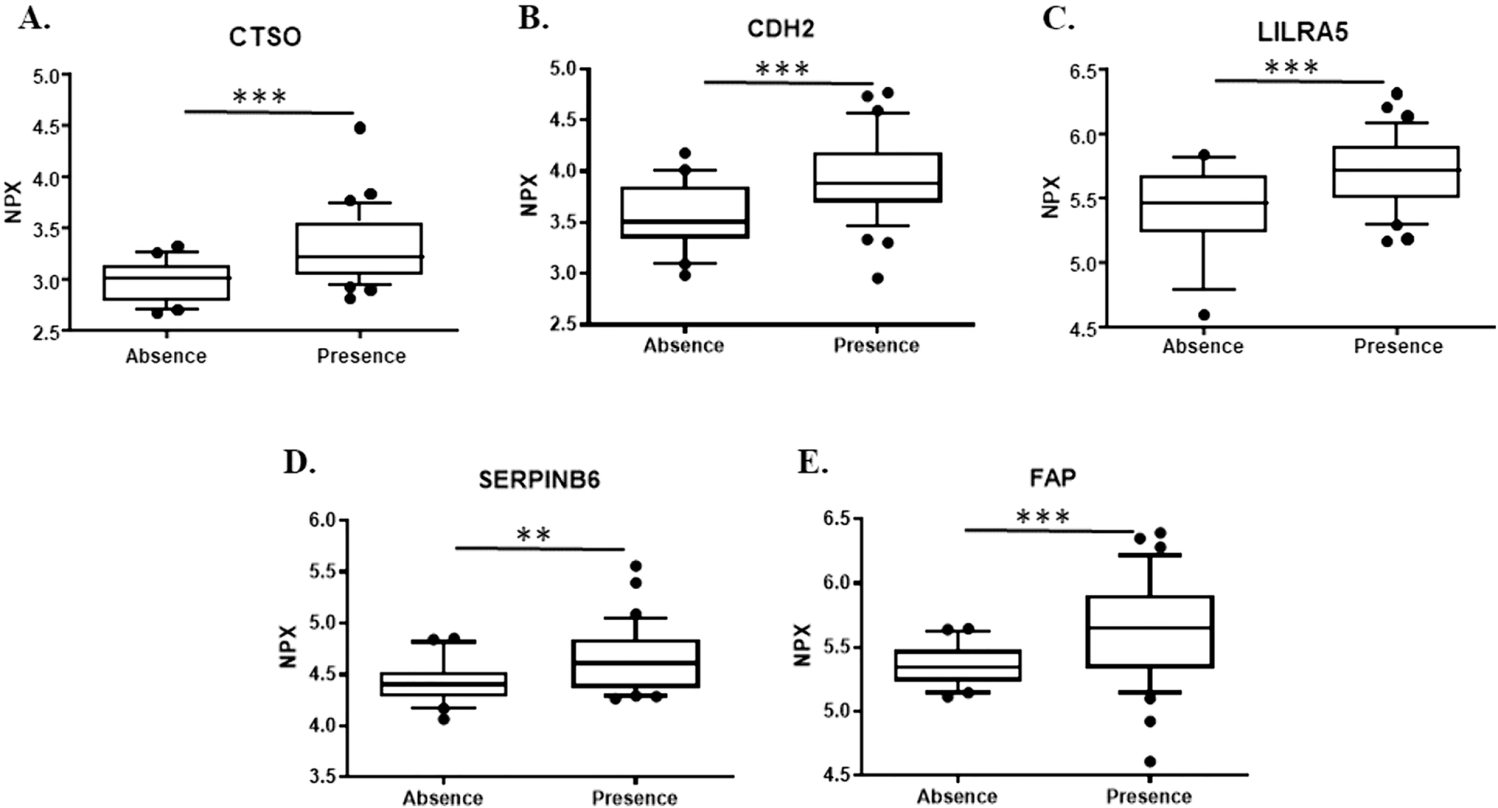
Serum protein expression for proteins under study in obese adolescents with and without steatosis. Box plots depict the differences in NPX values for individuals without steatosis (S0, *n* = 21; Absence) has been compared with those with steatosis (S1, S2, S3, *n* = 37; Presence). **A** CTSO protein, **B** CDH2 protein, **C** LILRA5 protein, **D** SERPINB6 protein and **E** FAP protein, Values were presented as median with their respective 10–90 percentiles. Differences were considered statistical significants at *p values* less than 0.05. *p < 0.05, **p < 0.01, ***p < 0.001

In individuals with liver steatosis compared to those without, an increase in mean NPX values from 2.99 ± 0.20 to 3.32 ± 0.35 was detected for CTSO (padj < 0.003), from 3.55 ± 0.32 to 3.94 ± 0.40 for CDH2 (padj < 0.007), from 5.41 ± 0.35 to 5.72 ± 0.28 for LILRA5 (padj < 0.021), from 4.42 ± 0.20 to 4.65 ± 0.31for SERPINB6 (padj < 0.034) and from 5.36 ± 0.16 to 5.68 ± 0.40 (padj < 0.007) for FAP, indicating an increased serum expression of certain proteins (Fig. 2).

Afterward, we query the Human Protein Atlas portal (HPA) (https://www.proteinatlas.org/), finding that CTSO cluster as a plasma protein with the highest mRNA expression in the liver, CDH2 shows high protein expression in the liver, kidney, adrenal gland, and heart muscle, LILRA5 in lymphoid tissue and bone marrow, SERPINB6 is enriched in adipose tissue and FAP in connective tissue with higher expression in gallbladder (see Fig. S2). Moreover, a protein–protein interaction (PPI) network was created for the five identified markers in NetworkAnalyst 3.0. PPI was constituted by two subnetworks. The main subnetwork, with a total of 79 nodes and 78 edges is CDH2 protein the component with the highest number of interactions. Then, a second subnetwork was constituted by five total interactors. Then, functional enrichment was performed for all the protein components of the PPI in the Reactome platform. Most of the enriched proteins were clustered in functional activities related to signaling transduction, integration of energy metabolism, cell–cell communication, as well as the immune system and coagulation pathways. (see Figure S3 and Table S3).

### Gender differences in the serum proteome of adolescents with obesity

**Fig. 3:**
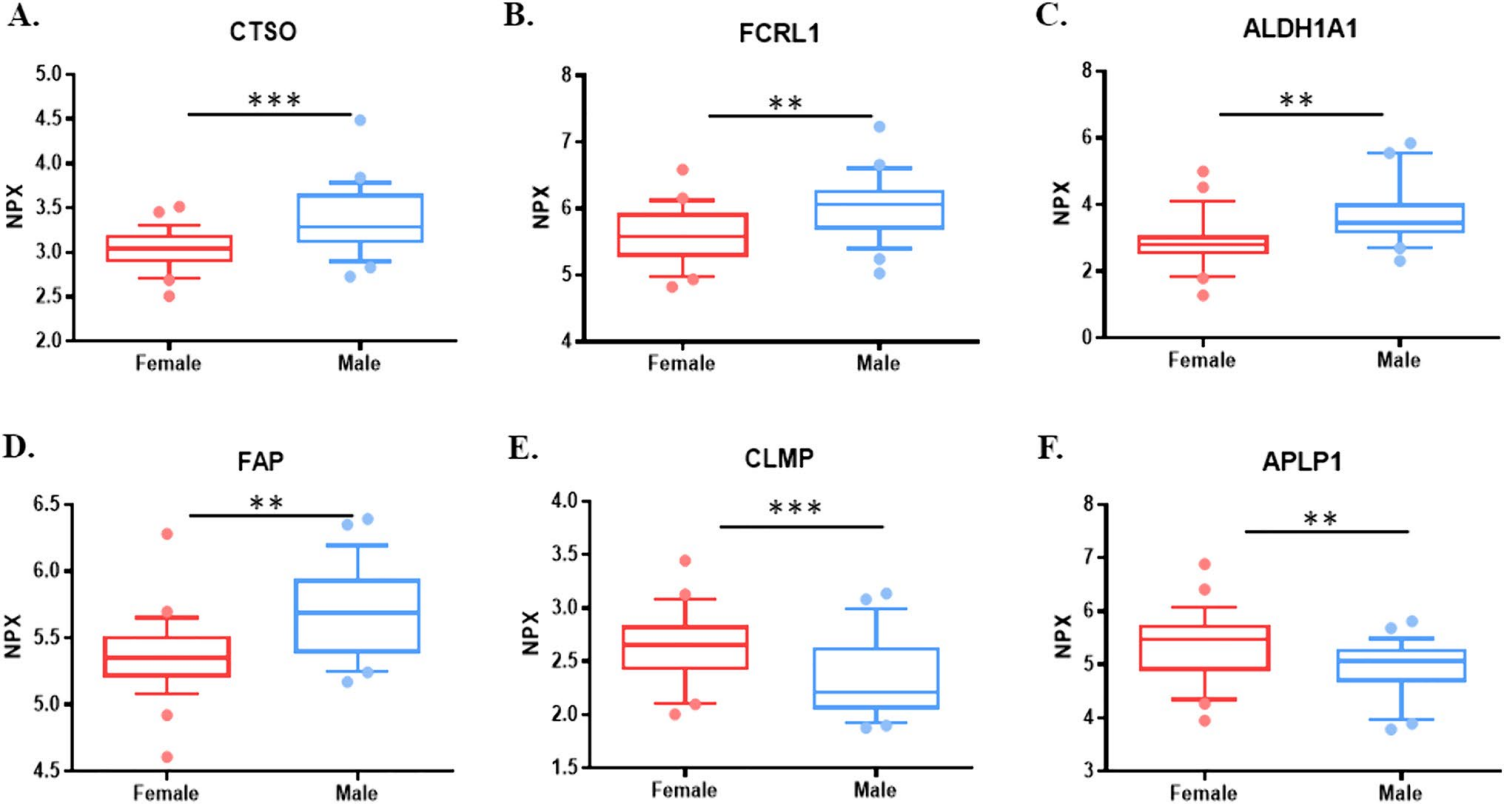
Serum protein expression differences according to gender for the proteins under study. Box plots show the variations according to gender, females (*n* = 28,) and males (*n* = 30). **A** CTSO protein, **B** FCRL1 protein, **C** ALDH1A1 protein, **D** FAP protein, **E** CLMP protein, and **F** APLP1 protein. Values were presented as median with their respective 10–90 percentiles. Differences were considered statistically significant at p-values less than 0.05. *p < 0.05, **p < 0.01, ***p < 0.001

After the initial proteome characterization of the serum samples according to the liver steatosis grade, we investigated the changes in the circulating proteome according to gender. The serum proteome of 28 female and 30 male subjects were compared. Our results show that 6 proteins of the 184 analyzed presented differences according to gender. Figure 3 shows the differences in normalized expression values for the proteins showing changes. CTSO, Fc receptor-Like 1 (FCRL1), Aldehyde Dehydrogenase 1 Family Member A1 (ALDH1A1), and FAP showed increased expression values in males (*p* = 0.001, *p* = 0.002, *p* = 0.002, and *p* = 0.002, respectively). On the other hand, CXADR Like Membrane Protein (CLMP), and Amyloid Beta Precursor Like Protein 1 (APLP1) serum expression were higher in females than males (p = 0.0007 and 0.002, respectively). Our results indicated that CTSO and FAP were affected not only by steatosis grade but also by the gender of the subject (Fig. 3 and Table S4).

### Correlation of steatosis grade with the clinic-biochemical parameters and serum protein expression

Multiple linear correlation analyses were performed to investigate the associations among the clinic-biochemical parameters, the serum proteome, and the steatosis grade. As shown in Table 3 and in the correlation plots (Figure S4) the most significant variables associated with steatosis were weight (ρ = 0.62, *p* < 0.0001), BMI (ρ = 0.50, *p* < 0.0001), waist circumference (ρ = 0.60, *p* < 0.0001), plasma insulin (ρ = 0.55, *p* < 0.0001), HOMA-IR (ρ = 0.55, *p* < 0.0001), GGT (ρ = 0.51, *p* = 0.0001) and the FLI (0.54, *p* < 0.0001). Moreover, among the proteins showing changes in their expression with steatosis, we observed CDH2 (ρ = 0.52, *p* < 0.001), CTSO (ρ = 0.44, *p* < 0.001), and LLRA5 (ρ = 0.45, *p* < 0.001) presenting the most significant correlations. Additional correlation analyses for steatosis degree and the rest of the parameters were performed when the cohort was also stratified by gender. Correlation matrix plots demonstrated, as an example, that CDH2 is associated with steatosis degree (rho = 0.46, *p* = 0.013), weight (rho = 0.43, *p* = 0.021), BMI (rho = 0.41, *p* = 0.030), insulin (rho = 0.38, *p* = 0.044), and HOMA-IR (rho = 0.41, *p* = 0.03), among other parameters in females (XY *n* = 28) and with steatosis degree (rho = 0.44, *p* = 0.017), insulin (rho = 0.41, *p* = 0.023), HOMA-IR (rho = 0.41, *p* = 0.025), and AST (rho = 0.65, *p* < 0.001), ALT (rho = 0.61, *p* < 0.001), GGT (rho = 0.38, *p* = 0.037); among others in males (XY *n* = 31) (Figure S5 A-C, and supplementary information Tables S5–S7).

**Table 3:** Significant correlations among biomolecular data and steatosis degree

XY *n* = 58	rho	R squared	*p-value*
Weight	0.62	0.39	< *0.0001*
Height	0.42	0.18	*0.001*
BMI	0.50	0.25	< *0.0001*
Waist circumference (cm)	0.60	0.33	< *0.0001*
FFM (%)	0.55	0.30	< *0.0001*
FM (kg)	0.45	0.20	*0.0004*
mREE (kcal)	0.48	0.23	*0.0001*
Insulin (mU/L)	0.55	0.30	< *0.0001*
HOMA-IR	0.55	0.30	< *0.0001*
Triglicerydes (mg/dL)	0.37	0.14	*0.004*
AST (U/L)	0.33	0.11	*0.013*
ALT (U/L)	0.37	0.13	*0.004*
GGT (U/L)	0.51	0.26	< *0.0001*
FLI	0.54	0.29	< *0.0001*
CTSO (NPX)	0.44	0.19	*0.0005*
SERPINB6 (NPX)	0.36	0.13	*0.005*
LLRA5 (NPX)	0.45	0.21	*0.0004*
ALDH1A1 (NPX)	0.38	0.14	*0.004*
CDH2 (NPX)	0.52	0.27	< *0.0001*
FAP (NPX)	0.37	0.14	*0.004*

*BMI* body mass index, *FFM* fat-free mass, *FM* fat mass, *mREE* measured resting energy expenditure, *HOMA-IR* homeostatic model assessment-insulin resistance, *AST* aspartate aminotransferase, *ALT* alanine aminotransferase, *GGT* gamma-glutamyl transferase, *FLI* fatty liver index. Expression values are reported as NPX median value (IQR). S0 steatosis grade 0: S0; steatosis grade 1, 2 and 3: S1-S2-S3

### Combination of serum proteins and clinical-biochemical parameters to distinguish liver steatosis

**Fig. 4:**
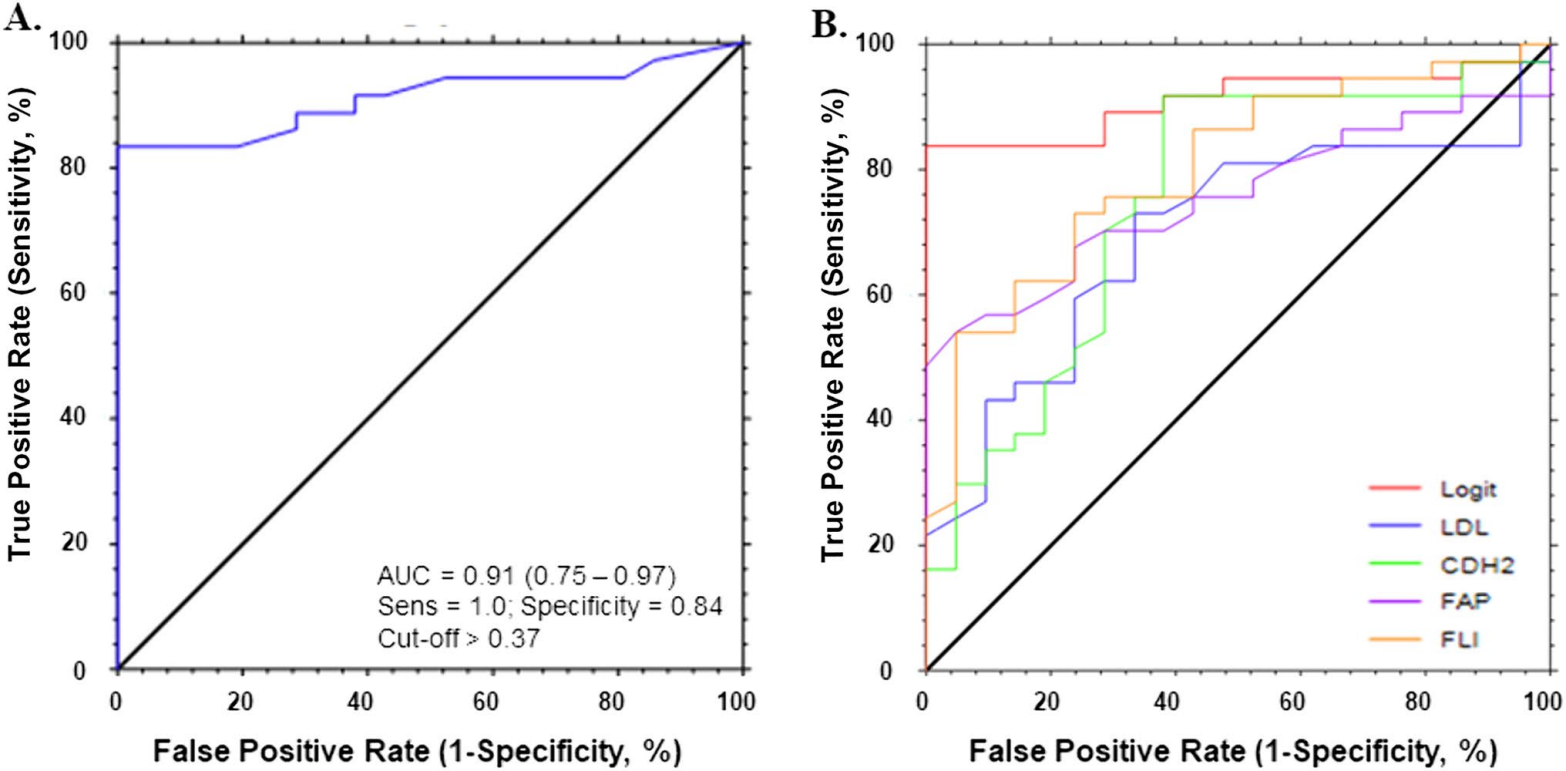
Receiver operating characteristic (ROC) curves for steatosis diagnosis in obese adolescents. **A** Logistic regression (logit) model combining the best 4 variables for steatosis outcome prediction. The logit model has the following equation: steatosis = -38.81 + 0.05*LDL + 3.21*CDH2 + 3.89*FAP, achieving a diagnostic performance with an area under the curve of 0.91 with a sensitivity of 100% and specificity of 84%. **B** ROCs for logit model and for each component of the model equation separately

The potential of the individuated proteins as biomarkers of liver steatosis was also assessed. We performed ROC analysis for each candidate marker, which showed a statistical correlation with the steatosis grade. AUC and cut-off values based on the Youden index, which maximizes sensitivity and specificity, are displayed in Table 4. Afterward, to obtain the best diagnostic performance for steatosis, a logistic regression model analysis (with forward selection with switching mode) considering all the variables under study was performed. The model includes 33 variables (32 numeric and gender as categorical). Continuous numerical variables were age, weight, height, BMI, waist-circumference, FFM, systolic PA, diastolic PA, REE, fasting glucose, insulin, HOMA-IR, triglycerides, total cholesterol, HDL, LDL, PCR, AST, ALT, GGT, platelets, NLR, APRI, FLI, CLMP, CTSO, FCRL1, SERPINB6, APLP1, LILRA5, ALDH1A1, CDH2 and FAP). The statistical analysis demonstrated that the best predictive model for steatosis diagnosis should include the four variables with the following equation: -38.81 + 0.05 * LDL + 3.21 * CDH2 + 3.89 * FAP achieving a diagnostic performance with AUC of 0.91 (95% CI 0.75–0.97), with a sensitivity of 100% and specificity of 84% at a cut-off value determined at > 0.37 (Fig. 4A). Moreover, the performance in diagnostic accuracy for steatosis using the FLI and each one of the components of the logit model was compared (Fig. 4B). Statistics associated with the figure reveal that the logit model achieved better diagnostic performance for liver steatosis than the components separately, particularly when comparing with the AUC of LDL (*p* < 0.0008), CDH2 (*p* < 0.0137) and FAP (0.0119), but not against FLI (*p* = 0.0845). In addition, Fig. S5 depicts the correlations between the variable outcome steatosis degree obtained with the logit model and several clinic-biochemical parameters for the overall cohort.

**Table 4:** Ranked list of the AUC values calculated for each of the biomarker candidates and the FLI

PROTEIN	UniProt	AUC	AUC SE	95% CI		cutoff value	(sens.)	(spec.)
CDH2	P19022	0.749	0.064	0.594	0.850	≥ 3.50	0.91	0.52
FAP	Q12884	0.748	0.059	0.607	0.843	≥ 5.70	0.54	0.95
CTSO	P43234	0.717	0.064	0.568	0.820	≥ 3.16	0.59	0.76
LILRA5	A6NI73	0.669	0.074	0.497	0.791	≥ 5.61	0.7	0.67
SERPINB6	P35237	0.640	0.071	0.480	0.758	≥ 4.61	0.49	0.86
FLI		0.801	0.06	0.650	0.890	≥ 89.2	0.73	0.76

## Discussion

Serum protein biomarkers for diagnostics and monitoring of liver steatosis appear useful to improve future noninvasive algorithms based on blood surrogate markers, particularly in monitoring subjects at high risk, such as young people with obesity. Proteomics has the potential to identify the best candidates to be included in the novel scores/algorithms. Since traditional proteomics requires a lot of sample pre-processing and data analysis, more user-friendly tools are being developed. Among others [33], PEA is one of the emergent and most robust available technology. It is multiplexed and requires only 1 µL of plasma sample. It is based on the PCR process, and data analysis is easy to perform with the software/pipelines available nowadays [34].

Currently, the most used non-invasive procedure for the diagnosis of MASLD is liver ultrasonography. In adolescents, clinicians have recently reported that supra iliac skinfold thickness US is useful in MASLD diagnosis [35]. Alternative, accurate, and inexpensive non-invasive diagnostic scores have the potential to monitor changes in the degree of hepatic steatosis. FLI, HIS, and ZJU indexes were initially validated for quantitative estimation of steatosis in adult population studies and have been recently externally validated in adolescents, showing that the three scoring systems had acceptable discrimination performance for steatosis (AUC > 0.7) [9]. We consider that the future of the diagnosis in MASLD will be improved by a combination of clinical parameters, plasma protein markers, and imaging methods, obtaining a reliable diagnosis, not only for steatosis but for the different stages of liver disease.

In this context, our study represents the first attempt to explore serum proteins associated with liver steatosis in young subjects with obesity using untraditional LC–MS proteomics. Our data demonstrated that five proteins of the 184 investigated in the proteomics analysis showed increased expression/abundance at the serum level with the accumulation of fat in the liver, according to ultrasonography. The candidates to be combined in future algorithms for steatosis are CTSO, CDH2, LILRA5, SERPINB6, and FAP. The strongest association with steatosis degree was for CDH2 plasma abundances. Interestingly, we have recently reported the association of this marker and CTSO protein plasma levels, among other proteins, with liver steatosis in subjects with severe obesity due to the rare genetic disease Prader-Willi syndrome [36]. HPA knowledge resource shows that CDH2 and CTSO proteins were among the five markers that were the most abundantly expressed at the protein level in the liver. CDH2 is a membrane protein with the highest abundance in the liver, kidney, adrenal gland, and heart muscle. CTSO is an enzyme with cytoplasmic expression in several different tissue types. Moreover, we observed that the functional enrichment of the components of the protein–protein interaction network obtained for the five interested markers demonstrated that these proteins are related to the integration of energy metabolism through its involvement in pathways of signal transduction such as glucagon-type ligand receptor, signaling by receptor tyrosine kinases, Class B/2 (Secretin family receptors) incretin synthesis, secretion, and inactivation and pathways involved in cell–cell communications such as CDH11 homotypic and heterotypic interactions, regulation of homotypic cell–cell adhesion, among others.

Additional evidence in the current literature supported the association of these markers with the pathophysiology/development of liver steatosis. Seda O. et al. (38) reported comparable transcriptome profiles of steatotic and non-steatotic transplanted grafts and the deregulation of clusters of genes involved in commonly affected pathways by MASLD, such as blood coagulation, cell redox homeostasis, glucagon-signaling pathways and among them also epithelial adherence junction signaling involving CDH2 network. Interestingly, the epigenetic variant rs11083271 (chromosome location 18:28,346,095) mapped in CDH2 has been associated with MASLD [37, 38]. In addition to our evidence on plasma samples from Prader-Willi syndrome, increased circulating plasma levels of CDH2 have been associated with diabetic nephropathy (41), and malignant bone and soft tissue tumors [39]. CTSO gene expression changes have been associated with microsome alterations in obesity in a transcriptome study (43), and increased plasma levels have been associated with the fat mass index (one of the best cardiometabolic disease risk predictors). Limited literature concerning LILRA5 changes associated with liver steatosis, MetS, or obesity, both at gene expression level or plasm abundance, has been reported [40, 41]. On the contrary, a strong knowledge of genetic association with HDL measurements, only second to the strong genetic relation of this gene with neurodegenerative diseases, is present in literature according to the OpenTargets platform [42–44]. An integrative transcriptome study of datasets from mouse and human liver tissues has associated SERPINB6, B1, and B9 increase with the development of HCC in MASLD [45]. Of our individuated proteins, FAP is the most well-known, according to current literature, for its involvement in MASLD. FAP was reported initially as a marker of hepatic stellate cell activation with increased intrahepatic expression correlating with the fibrosis stage in HCV patients [46]. Moreover, FAP plasma abundance changes have been associated with liver fibrosis not only in virus-related cirrhosis but also in non-cirrhotic patients with MASLD in diabetes and obesity [47] [48]. Interestingly, FAP has been recently tested as a pharmacological target not only for fibrosis but also as a candidate for anti-diabetic and anti-steatotic therapies since it is involved in the regulation activities of FGF21(a stress-induced hormone with potent anti-obesity, insulin-sensitizing, and hepatoprotective properties) [49–51].

In this exploratory study, after identifying the potential markers, we tested the discriminatory performance of each candidate for the diagnosis of steatosis in adolescents. Individually, CDH2 showed the best performance for the desired outcome in the study cohort, comparable to the obtained for the FLI. Afterward, all the clinic-biochemical parameters and candidate markers were tested using a logistic regression model to improve the diagnostic performance through the combination of the best four associated variables. The resulting statistical model, which included CDH2, FAP, and LDL variables, achieved an optimal diagnostic performance of AUC of 0.91 (95% CI: 0.75–0.97) with a sensitivity of 100% and specificity of 84%, underlying the relevance of the combination of novel parameters to establish the future blood surrogates’ indexes with the most reliable parameters for the minimally invasive diagnosis of steatosis in MASLD, clinical management and prognosis.

In the upcoming years, the MASLD diagnostic scores based on surrogate markers will be substantially improved, and clinical diagnostic models powered by AI including more complex datatypes will be validated to predict the stage of the disease in a more accurate, easy, non-invasive, and robust manner. Currently, the fatty liver diagnostic algorithms which were initially tested and validated in adult populations, are being also tested and validated in pediatric cohorts. Wan et al. [9] tested the discrimination capacity of FLI, HIS, and ZJU indexes in 899 adolescents and compared them with supra iliac skinfold thickness by the US obtaining better discrimination for MASLD in males with the following AUCs (FLI 0.82, HIS 0.83, ZJU 0.83 and US supra iliac thickness: 0.88). Shi [52] et al. evaluated the diagnostic performance in 1845 children obtaining AUCs of 0.96, 0.96, and 0.77 for HSI, ZJU index, and TyG index (triglyceride-glucose index), respectively.

Our study has some limitations. Firstly, the diagnosis of steatosis was based on a liver echography, which is less sensitive for detecting minor hepatic steatosis than biopsy; however, liver US is the recommended and accepted non-invasive test for assessing suspected MASLD or for screening studies in the general population. Secondly, the sample size of our cohort was relatively small. Still, the associations with steatosis for three of the five individuated markers were also evident in the Prader-Willi cohort, emphasizing the potential of these proteins as biomarkers for fatty liver and encouraging further investigation in larger cohorts of pediatric and adult subjects. Lastly, comparison with additional fatty liver scoring systems based on surrogate markers (HIS, ZJU) and diagnosis with other imaging methods (proton magnetic resonance spectroscopy (^1^H-MRS) liver evaluation) or the gold standard liver biopsy was not performed.

The current diagnostics of hepatic steatosis in pediatric subjects based on US and surrogate indexes need additional improvements, and this can be achieved with a future combination of them in novel algorithms accounting for additional protein markers such as CDH2 and FAP.

## Supplementary Information

Below is the link to the electronic supplementary material.

Supplementary file1 (DOCX 1877 KB)

Supplementary file2 (XLSX 150 KB)

## Data Availability

Raw data will be uploaded on www.zenodo.org immediately after the publication of the manuscript and they will be available upon a reasonable request to the corresponding author

## Abbreviations

MASLD: Metabolic dysfunction associated with steatotic liver disease
MASH: Metabolic dysfunction associated with steatohepatitis
FLI: Fatty liver index
HSI: Hepatic steatosis index
ZJU: Zhe Jiang university index
APRI: Aspartate aminotransferase to platelet ratio index
PEA: Proximity extension assay
NPX: Normalized Protein eXpression
PPI: Protein protein interaction
CTSO: Cathepsin O protein
CDH2: Cadherin 2 protein
LILRA5: Leukocyte immunoglobulin-like receptor subfamily A member 5 protein
SERPINEB6: Serpin B6 protein
FAP: Prolyl endopeptidase protein
