# Supplementary material for: Serum proteome signatures associated with liver steatosis in adolescents with obesity
Source: J Endocrinol Invest. 2024 Jul 17;48(1):213–25. doi: 10.1007/s40618-024-02419-x (PMC11729140; doi:10.1007/s40618-024-02419-x)
Supplement: Supplementary file 1 — Supplementary file1 (DOCX 1877 KB) [file 40618_2024_2419_MOESM1_ESM.docx]

***Supplementary Information***

**Serum proteome signatures associated with liver steatosis in adolescents with obesity**

**Pablo J. Giraudi^1^(†), Devis Pascut^2^(†), Cristina Banfi^3^, Stefania Ghilardi^3^, Claudio Tiribelli^1,2^, Adele Bondesan^4^, Diana Caroli^4^, Alessandro Minocci^5^, Alessandro Sartorio^4,6^**

^1^Fondazione Italiana Fegato - ONLUS, Metabolic Liver Disease Unit, Trieste, Italy

^2^Fondazione Italiana Fegato - ONLUS, Liver Cancer Unit, Trieste, Italy

^3^Unit of Functional Proteomics, Metabolomics, and Network analysis, Centro Cardiologico Monzino, IRCCS, Milan, Italy

^4^Istituto Auxologico Italiano IRCCS, Experimental Laboratory for Auxo-endocrinological Research, Piancavallo-Verbania, Italy

^5^Istituto Auxologico Italiano IRCCS, Division of Metabolic Diseases, Piancavallo-Verbania, Italy

^6^Istituto Auxologico Italiano IRCCS, Experimental Laboratory for Auxo-endocrinological Research, Milan, Italy

(†) These authors contributed equally to this work and shared the first authorship

*** Correspondence:**Pablo J. Giraudi pablo.giraudi@fegato.it

**ORCID:** https://orcid.org/0000-0003-2852-6648

**Total supplementary figures: 5**

**Total supplementary tables: 7 (in xlsx format)**

**Page 3.** Figure S1 A-E, Figure S2 A-B

**Page 4.** Figure S2 C-E

**Page 5.** Figure S2 F-G, Figure S3

**Page 6.** Figure S4

**Page 7.** Figure S5 A

**Page 8.** Figure S5 B

**Page 9.** Figure S5 C

**Page 10, 11.** Python scripts

**Supplementary tables (xlsx spreadsheets):**

**Table S1.** Olink 92 proteins Metabolism and Cardiometabolic panels

**Table S2.** Olink Metabolism and Cardiometabolic statistical data for steatosis comparison analysis

**Table S3.** Functional enrichment data from Reactome platform

**Table S4.** Olink Metabolism and Cardiometabolic panels for female/male comparison analysis

**Table S5.** Female correlation matrix (correlation coefficients and associated p-values)

**Table S6.** Male correlation matrix (correlation coefficients and associated p-values)

**Table S7.** Overall cohort correlation matrix (correlation coefficients and associated p-values)


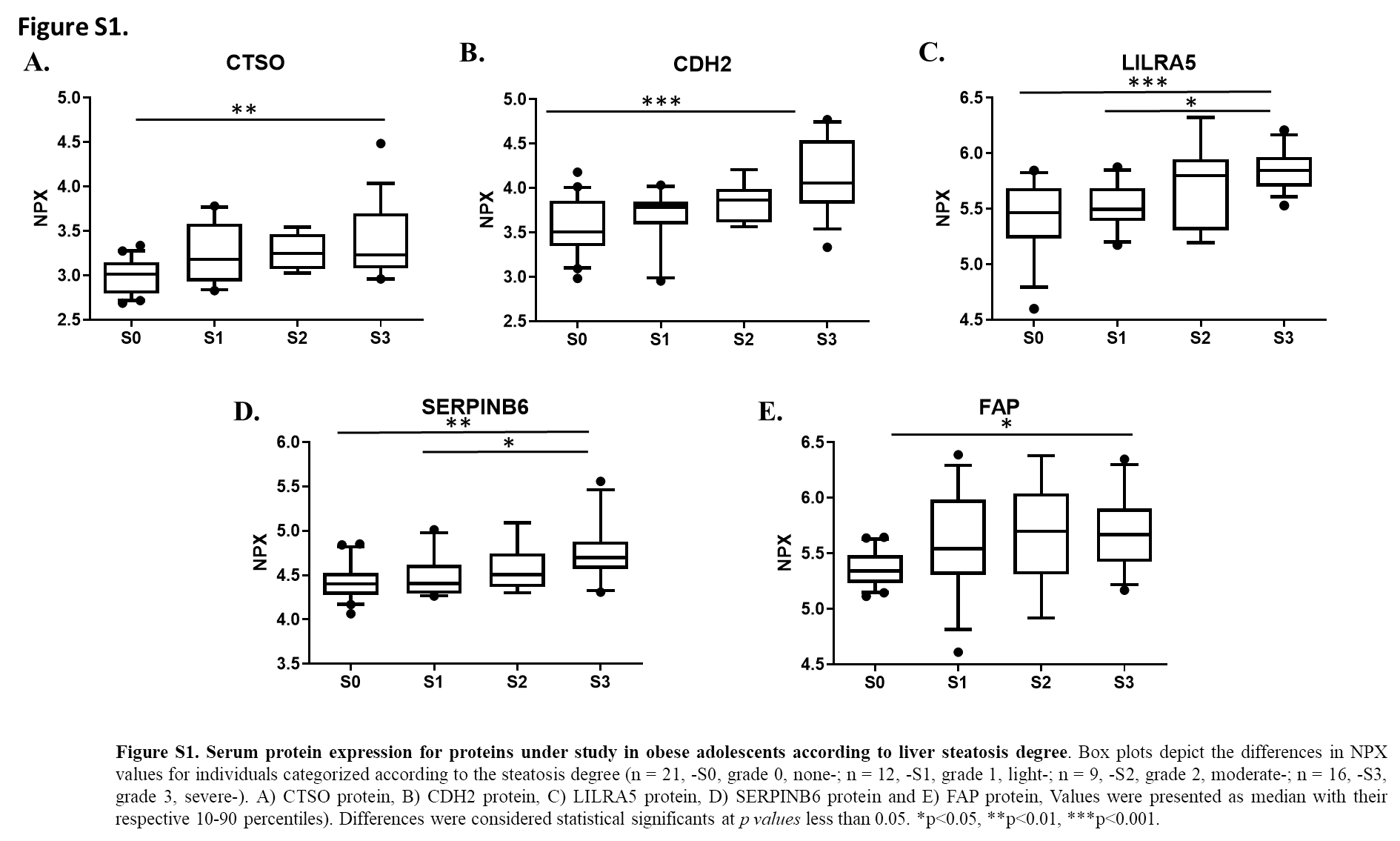


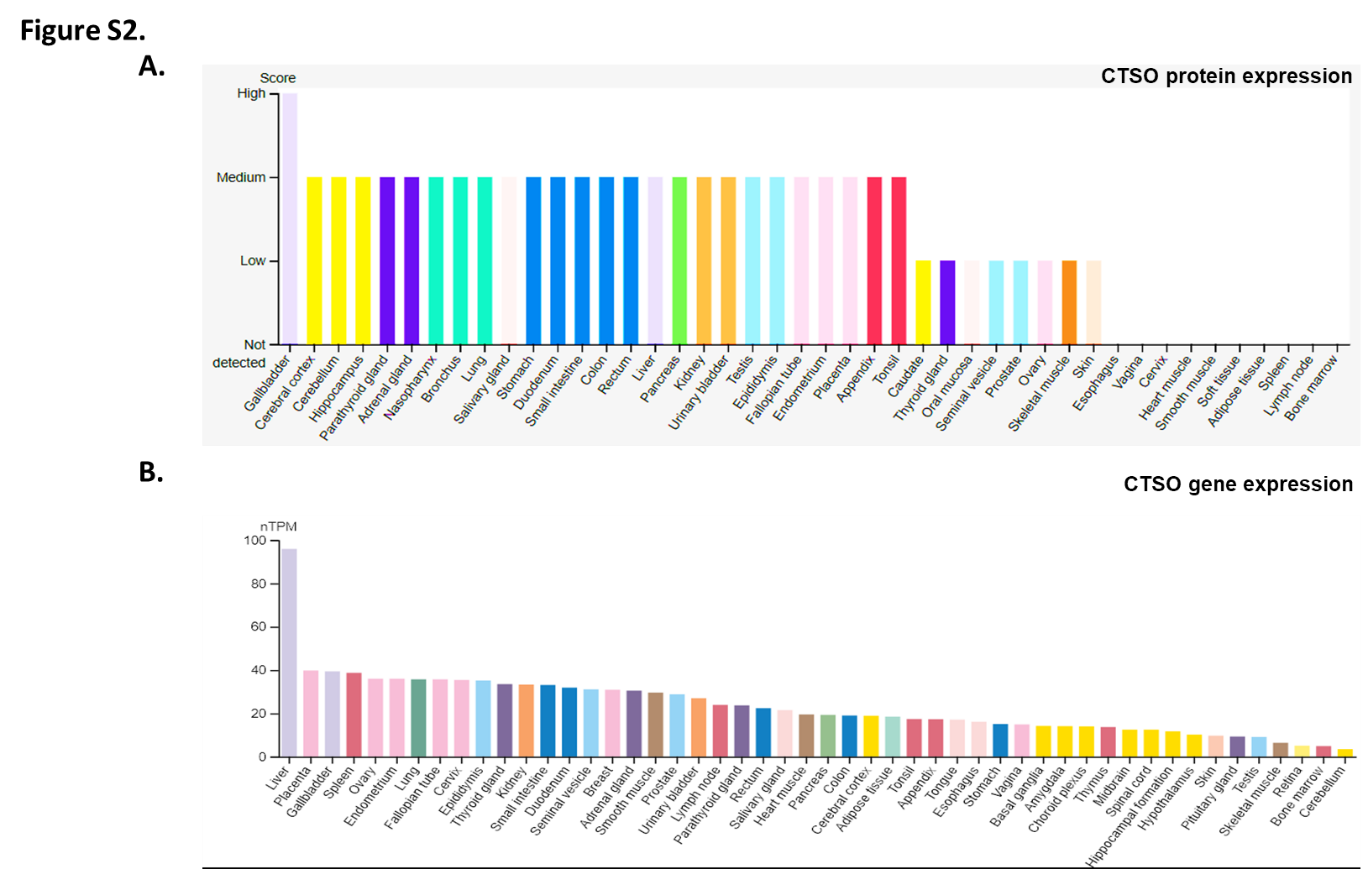


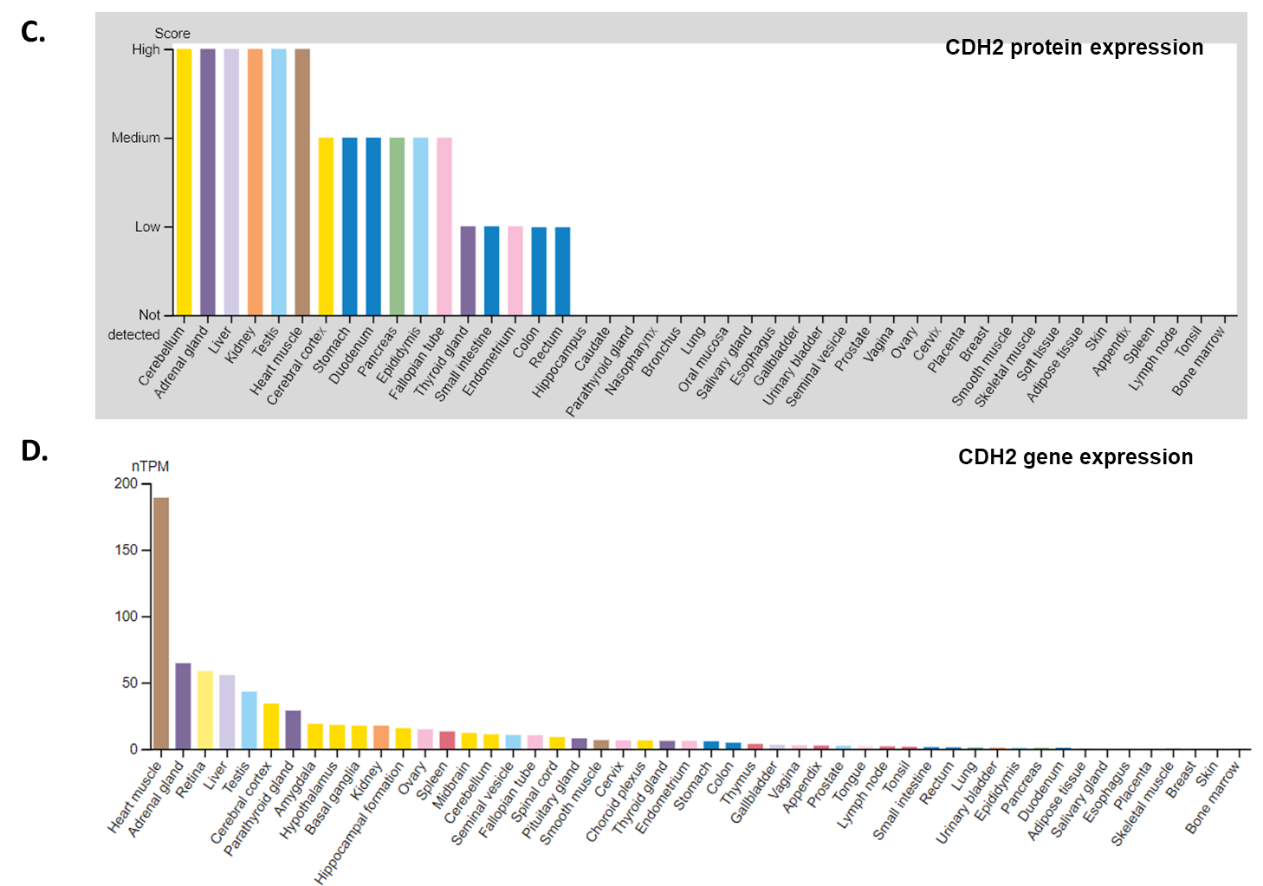


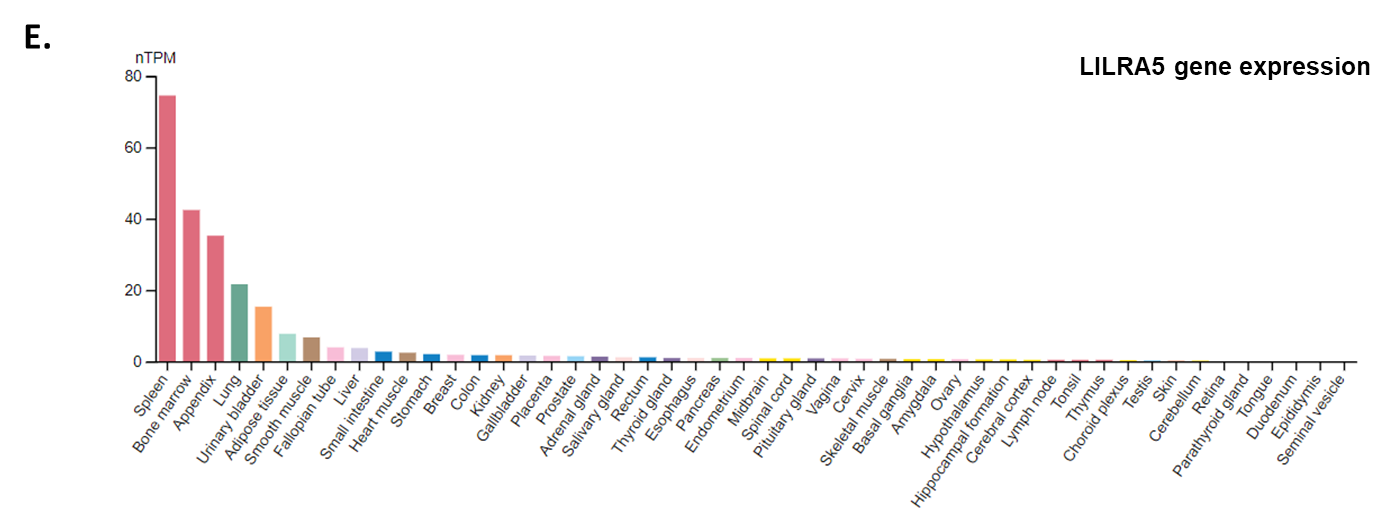


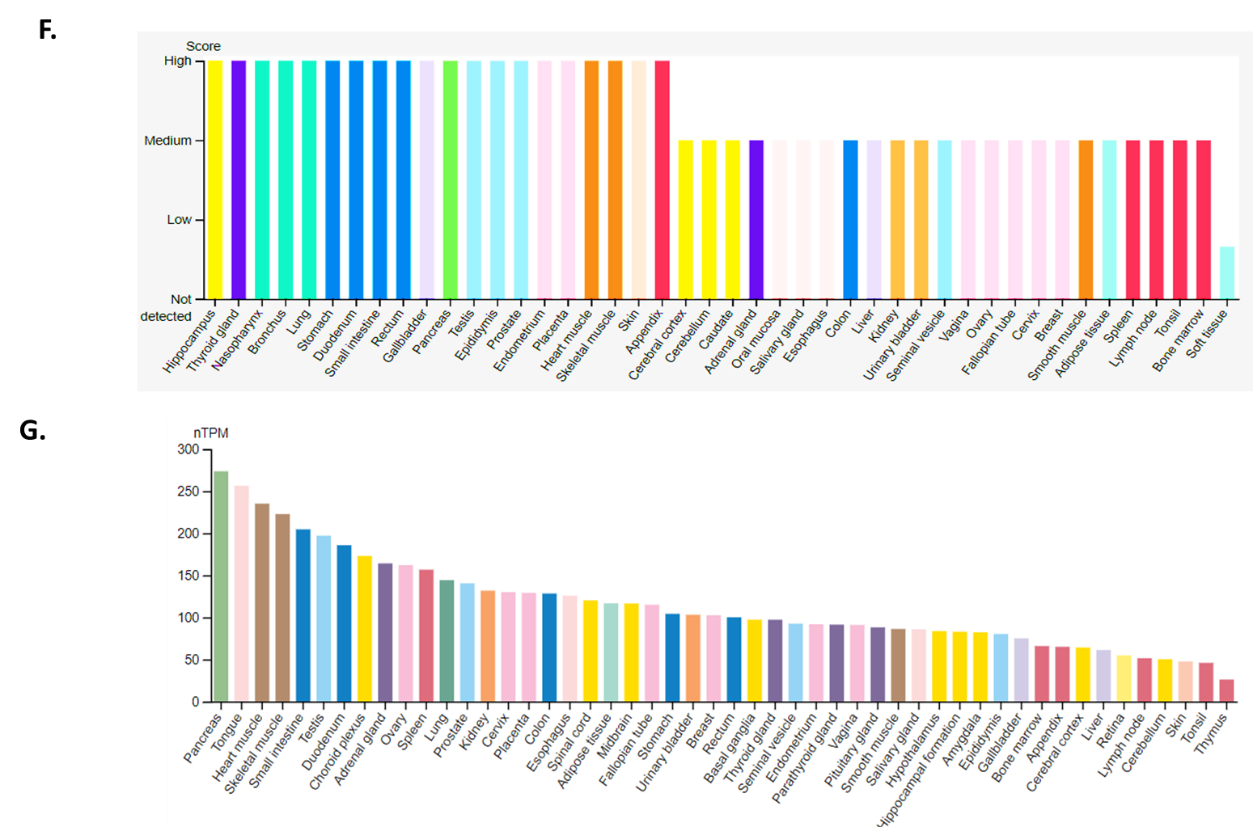


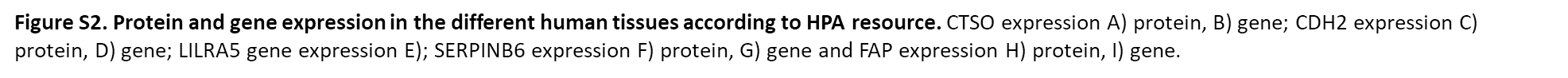


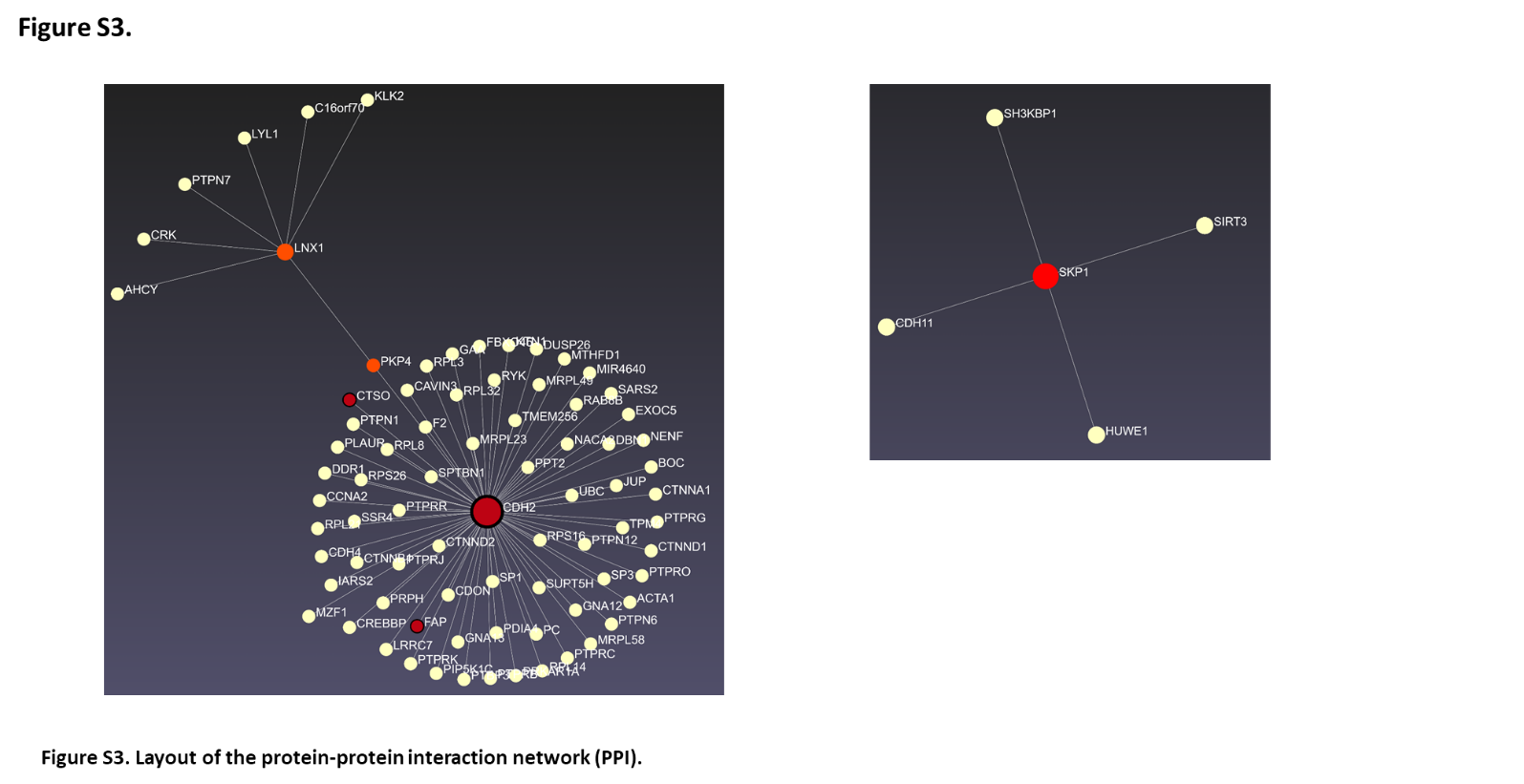


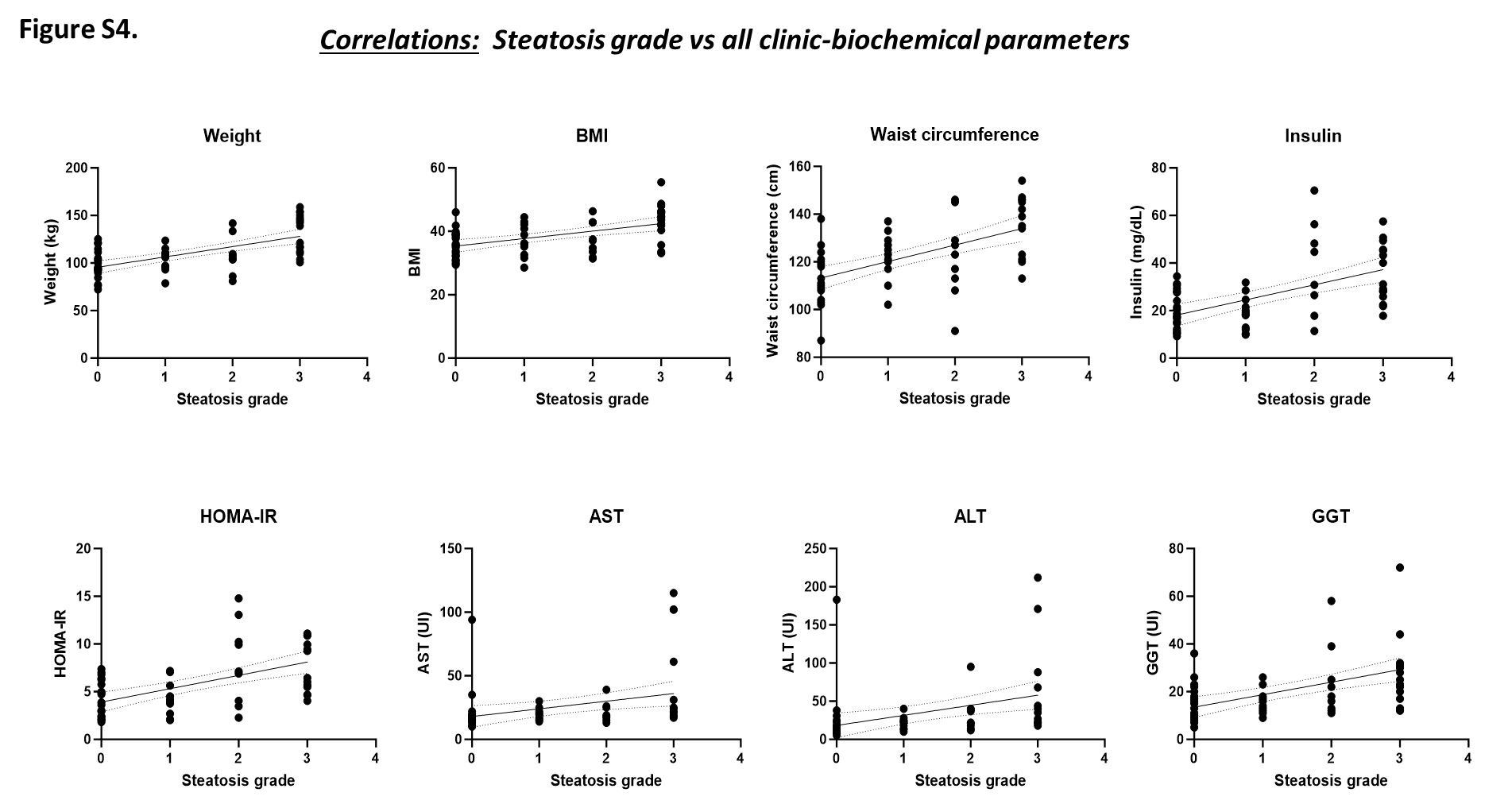


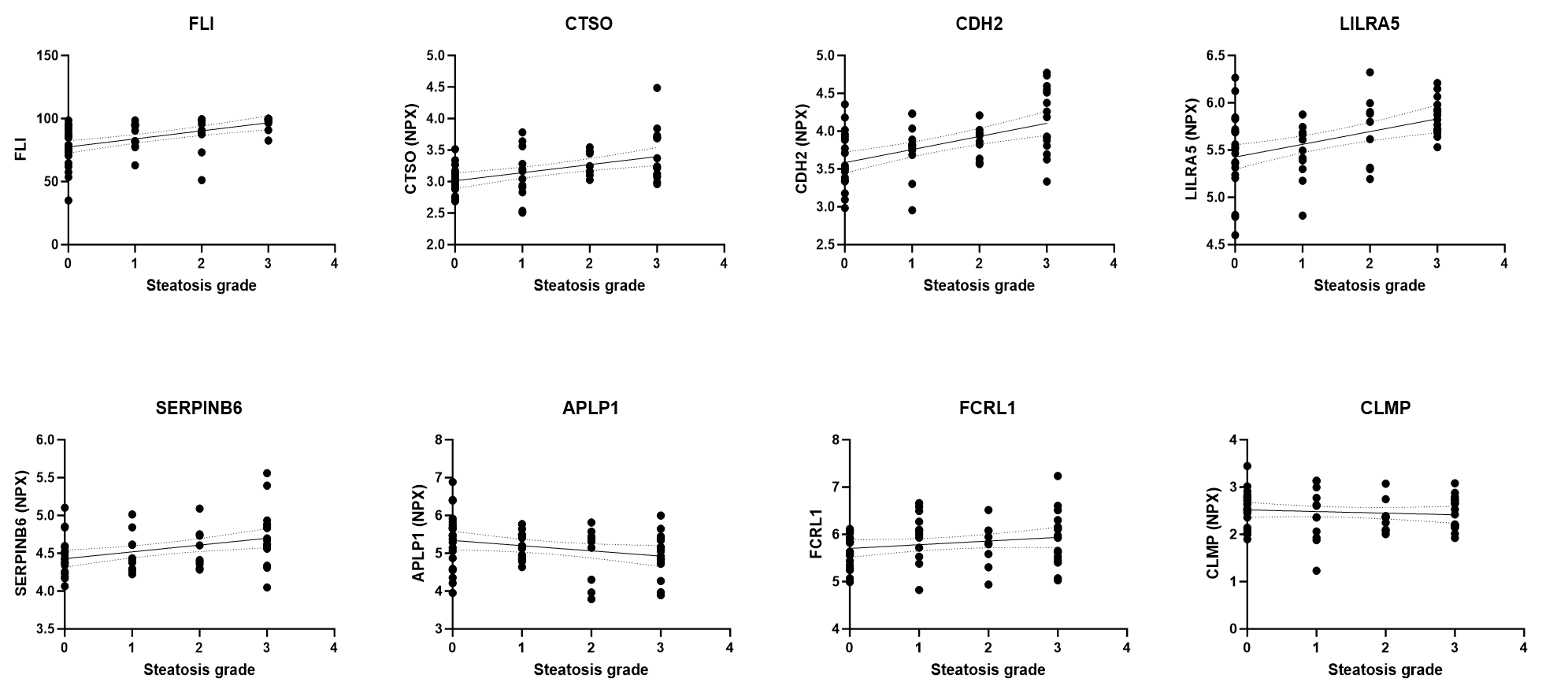


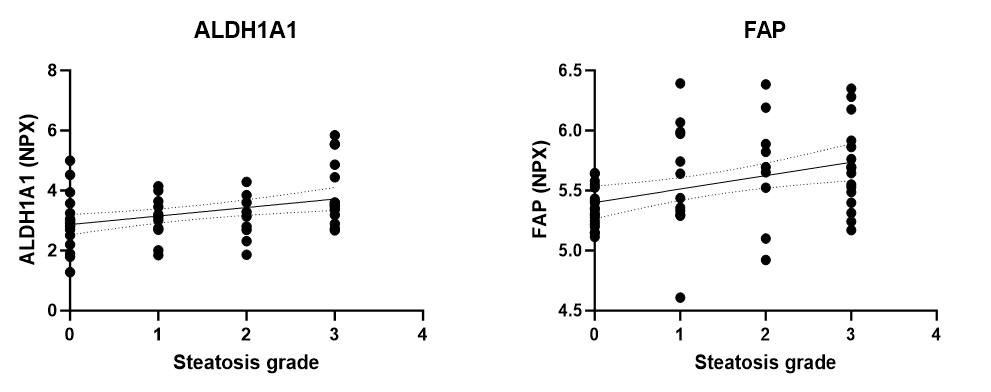


**Figure S5**

**
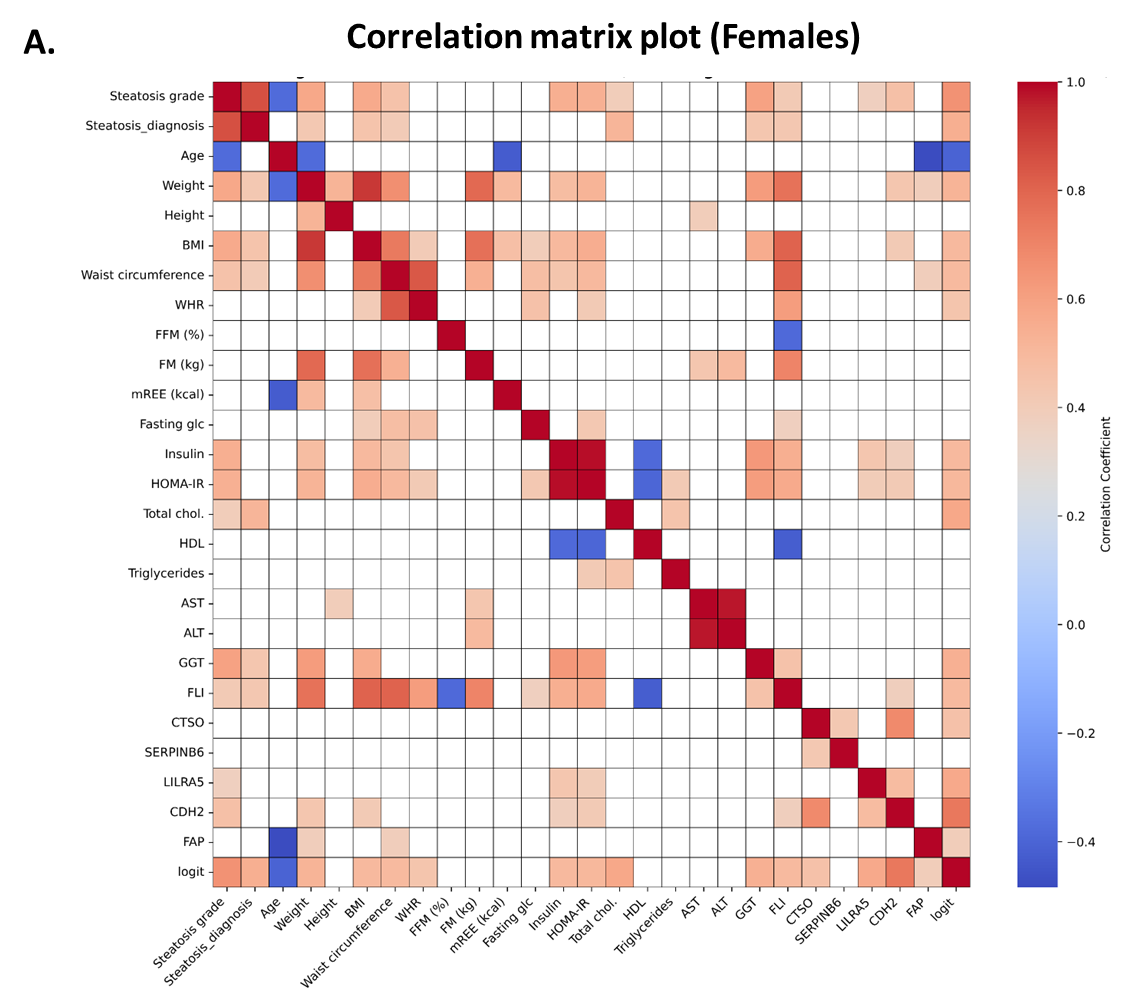
**

**
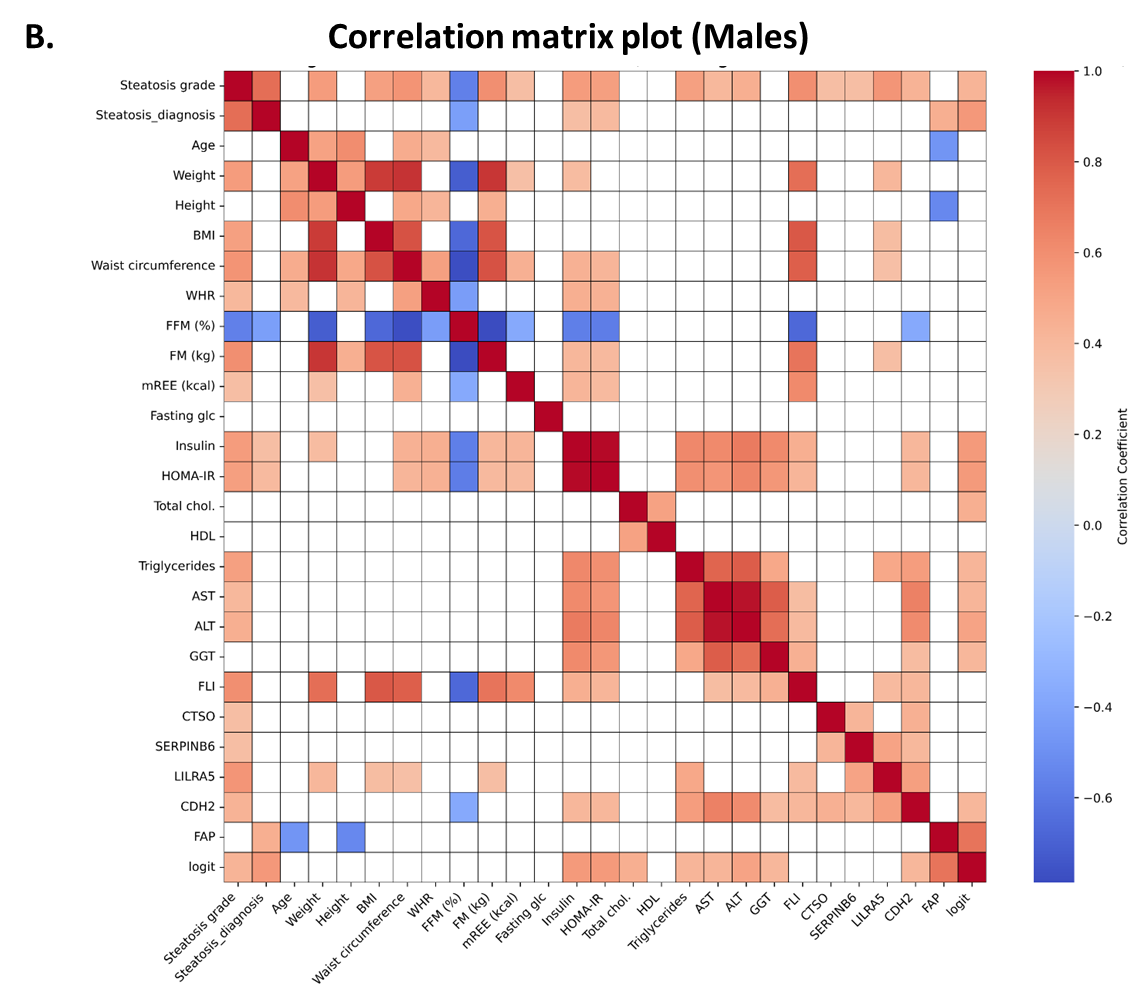
**

**
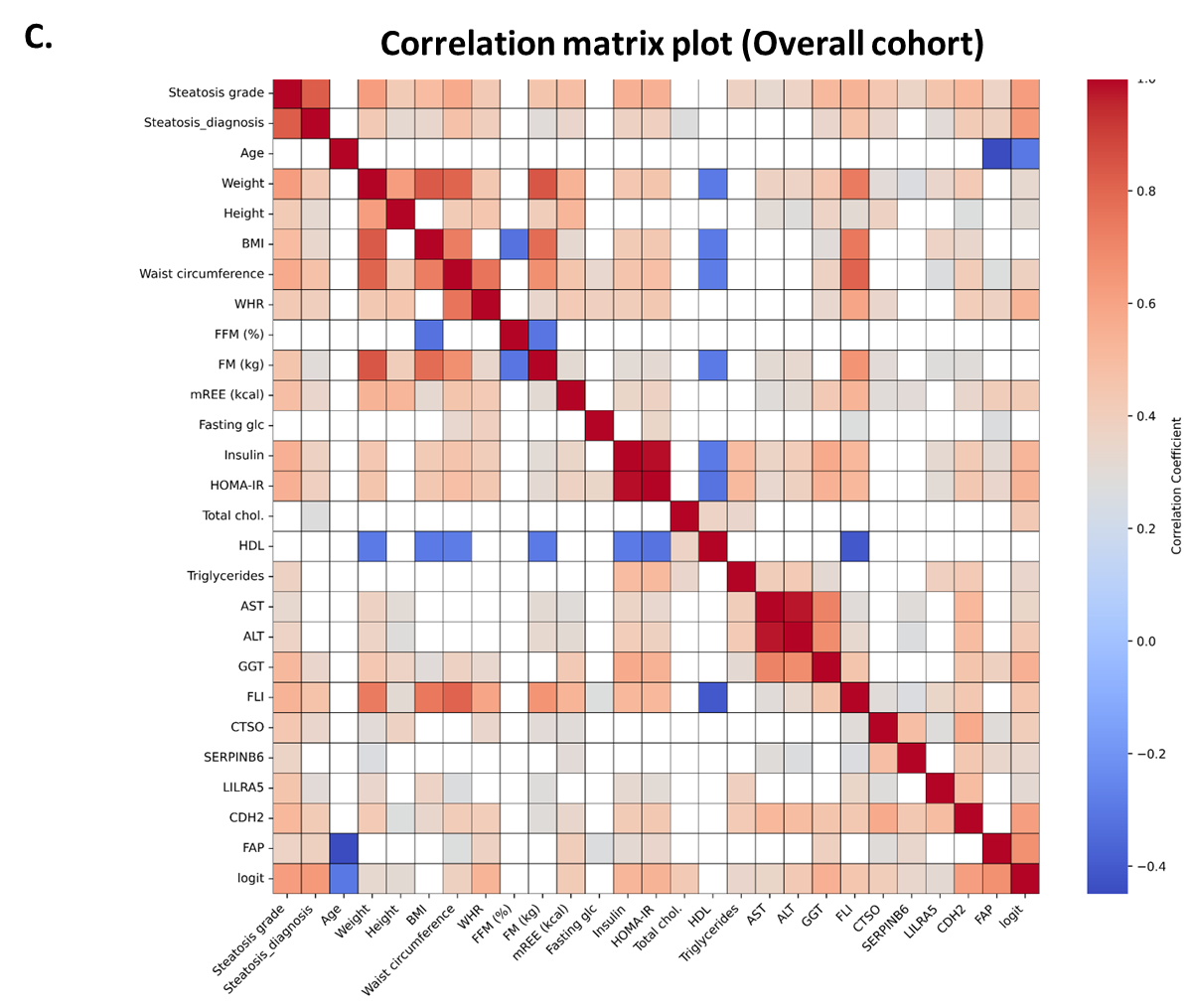
**

**Figure S5. Correlation matrix plots for clinical-biochemical parameters, individuated markers and logit model.** A) Female correlations, B) Male correlations, and C) Overall cohort correlations. The intensity of the color for each square in the matrix is proportional to the correlation coefficient value. White squares indicate non-significant associations.

**Code scripts to obtain Volcano and correlation matrix plots using Python**

***# Generation of volcano plots using Python 3.12***

import pandas as pd

# Load the new dataset

file_path = 'Volcano S0vsS123_final_full_ttest_results.csv'

df = pd.read_csv(file_path)

# Display the first few rows of the dataframe to understand its structure

print(df.head())

import numpy as np

import matplotlib.pyplot as plt

# Calculate the negative log10 of the Adjusted_pval

df['-log10(Adjusted_pval)'] = -np.log10(df['Adjusted_pval'])

# Define the threshold for statistical significance

significance_threshold = 0.05

# Identify significant and non-significant points

significant_mask = df['Adjusted_pval'] < significance_threshold

# Create the volcano plot with adjusted Y-axis scaling

plt.figure(figsize=(10, 6))

# Plot non-significant points in grey

plt.scatter(df.loc[~significant_mask, 'estimate'], df.loc[~significant_mask, '-log10(Adjusted_pval)'],

color='grey', alpha=0.5)

# Plot significant points in red or green

plt.scatter(df.loc[significant_mask, 'estimate'], df.loc[significant_mask, '-log10(Adjusted_pval)'],

c=np.where(df.loc[significant_mask, 'estimate'] > 0, 'green', 'red'),

alpha=0.5)

plt.axhline(y=-np.log10(significance_threshold), color='grey', linestyle='--')

plt.xlabel('Parameter Estimate')

plt.ylabel('-log10(Adjusted_pval)')

plt.title('Volcano Plot with Highlighted Significant Genes')

plt.ylim(0, 3)

plt.yticks(np.arange(0, 4, 1))

plt.show()

***# Generation of correlation matrix plots using Python 3.12***

from scipy import stats

import numpy as np

import pandas as pd

import seaborn as sns

import matplotlib.pyplot as plt

# Load the new dataset

df_new = pd.read_csv('your_dataset.csv')

# Remove non-numeric columns

numeric_df_new = df_new.select_dtypes(include=[np.number])

# Define significance threshold

significance_threshold = 0.05

# Calculate correlation coefficients and p-values including Steatosis grade as a continuous variable

corr_matrix_new = numeric_df_new.corr()

pval_matrix_new = numeric_df_new.corr(method=lambda x, y: stats.pearsonr(x, y)[1])

# Create a mask for significant correlations

significant_mask_new = pval_matrix_new < significance_threshold

# Apply the mask to the correlation coefficients

significant_corr_matrix_new = corr_matrix_new.where(significant_mask_new, other=np.nan)

# Maintain the diagonal for self-association

significant_corr_matrix_with_diag_new = significant_corr_matrix_new.copy()

np.fill_diagonal(significant_corr_matrix_with_diag_new.values, 1)

# Plot the correlation matrix for the significant coefficients with the original color palette and diagonal maintained

plt.figure(figsize=(14, 12))

sns.heatmap(significant_corr_matrix_with_diag_new, annot=False, cmap='coolwarm', cbar_kws={'label': 'Correlation Coefficient'}, linewidths=0.5, linecolor='black')

plt.title('Correlation Matrix of Significant Correlation Coefficients (Including Steatosis Grade as Continuous Variable)', fontsize=16)

plt.xticks(rotation=45, ha='right')

plt.yticks(rotation=0)

plt.show()
